# Lateral Drop-Weight Impact Response of SRC Columns with Built-In L-Shaped Steel: Role of Impact Velocity, Axial Compression Ratio, and Stirrup Spacing

**DOI:** 10.3390/ma19081489

**Published:** 2026-04-08

**Authors:** Yiwei Tang, Liu Yang, Yali Feng, Ni Zhang, Jixiang Li, Lei Zeng

**Affiliations:** 1 Yangtze University College of Arts and Sciences, Jingzhou 434020, China; 201872475@yangtzeu.edu.cn (L.Y.); f_yali@126.com (Y.F.); zhangni0212@163.com (N.Z.); jixiangli@whpu.edu.cn (J.L.); 2 School of Civil and Hydraulic Engineering, Huazhong University of Science and Technology, Wuhan 430074, China; 3 College of Civil Engineering and Architecture, Anhui Polytechnic University, Wuhu 241000, China; zenglei@yangtzeu.edu.cn; 4 Engineering Research Center of Anhui Green Building and Digital Construction, Anhui Polytechnic University, Wuhu 241000, China

**Keywords:** L-shaped SRC column, lateral impact, drop-weight impact test, dynamic response, axial compression ratio, transverse reinforcement detailing

## Abstract

**Highlights:**

**What are the main findings?**

- Seven drop-weight tests characterized L-shaped SRC columns under lateral impact.
- Impact velocity governed force demand, deformation growth, and energy dissipation.
- Intermediate stirrup spacing (s = 150 mm) gave the most balanced overall response within the investigated range.
- Axial compression enhanced resistance and reduced residual deformation.
- The embedded L-shaped steel section preserved load-path continuity after severe concrete crushing.

**What are the implications of the main findings?**

- The tests provide benchmark data for asymmetric SRC columns under lateral impact.
- The results clarify the roles of axial compression and transverse confinement.
- The findings support preliminary assessment and transverse detailing under severe lateral impact.

**Abstract:**

L-shaped steel-reinforced concrete (SRC) columns are commonly used as edge and corner members in bridge piers and high-rise buildings. However, systematic experimental evidence on their dynamic behavior and detailing effects under lateral impact remains limited. This study presents a parametric drop-weight impact program on seven SRC columns with built-in L-shaped steel sections. The effects of impact velocity (v), axial compression ratio (n = 0–0.2), and stirrup spacing in the non-densified region (s = 100–200 mm) were examined in terms of damage evolution, impact-response indices (Fmax, Fave, Δmax, Δres, T), and energy absorption efficiency (η = E_ab_/E). The results show that impact velocity was the dominant parameter governing both response amplitude and damage severity. Increasing v from 7.67 to 9.90 m/s increased Δmax and Δres by 92.6% and 144.3%, respectively, while η increased from 60.7% to 74.6%. Within the investigated range, axial compression improved resistance and suppressed residual deformation. As n increased from 0 to 0.2, Fmax and Fave increased by 17.5% and 30.4%, respectively, whereas Δres decreased by 32.1%. The effect of stirrup spacing on η was non-monotonic. The intermediate spacing (s = 150 mm) yielded the highest energy absorption ratio (60.7%) and the most balanced overall response among the tested cases, rather than representing a definitive optimum. No global buckling of the embedded steel section was observed, and all specimens maintained overall structural integrity under high-energy impact. These results provide experimental evidence for the response assessment and preliminary transverse detailing of asymmetric SRC columns under lateral impact.

## 1. Introduction

Civil infrastructure may be exposed to accidental lateral collisions during service, particularly from vehicles or vessels. Although such events are infrequent, they may cause severe local damage and, in extreme cases, progressive collapse of the structural system [1,2]. Edge and corner columns in bridges, port structures, and high-rise buildings are especially vulnerable because they are directly exposed and may experience high-energy, short-duration lateral loads. Improving the impact resistance of these critical vertical members is therefore an important issue in structural safety and resilience.

Steel-reinforced concrete (SRC) members combine high load-carrying capacity with good ductility and seismic performance, and are widely used in major structural components [3,4]. According to the configuration of the embedded steel section, SRC columns may be classified as symmetric or asymmetric. In practice, L- and T-shaped sections are often used in edge and corner columns to satisfy non-uniform boundary conditions and directional demand. Compared with symmetric sections, asymmetric SRC columns exhibit direction-dependent stiffness and strength. Under lateral impact, this anisotropy may lead to more complex force-path redistribution, flexure-shear interaction, and energy-dissipation behavior.

The lateral impact behavior of reinforced concrete (RC) columns has been extensively studied [5,6,7,8,9,10,11]. Existing work shows that the response depends strongly on axial compression ratio, longitudinal reinforcement, and transverse reinforcement detailing [5,6,7,8]. Improved transverse confinement helps restrain damage and deformation [6,7,8,9]. Member geometry and impact scenario also play important roles [10,11]. Taken together, RC columns under lateral impact remain susceptible to brittle shear-type failure. Their impact resistance depends strongly on axial load level and detailing, while conventional reinforcement alone is often insufficient to produce a substantial improvement.

Concrete-filled steel tube (CFST) members have therefore been widely investigated as an alternative impact-resisting solution to conventional RC columns under impact [12,13,14,15,16]. Their improved performance is generally attributed to external steel-tube confinement, which stabilizes the concrete core and sustains composite action during transient loading [16,17,18]. Previous studies have shown that the impact response of CFST members is governed mainly by confinement level, steel strength, and impact severity [17,18,19,20]. By contrast, the influence of concrete strength is often less pronounced [21,22,23]. The full-scale validation of impact behavior under more realistic scenarios remains limited [24,25]. This mechanism differs from that of SRC columns, where an internal steel skeleton provides load-path continuity and confinement from within the section.

Research on SRC members under impact has increased in recent years, but the available evidence still focuses mainly on symmetric sections or selected strengthening schemes [26,27,28]. Existing studies show that SRC members generally outperform conventional RC members in impact resistance and energy dissipation [29,30,31,32], and that the embedded steel skeleton plays a decisive role in sustaining resistance after local concrete damage [30,31,32]. However, most of these findings were obtained for symmetric SRC sections or for members with a mechanically balanced steel arrangement.

For asymmetric SRC columns, the situation is less clear. In practice, L- and T-shaped columns contain an intrinsically unbalanced steel layout, which introduces direction-dependent stiffness and strength. Under lateral impact, this asymmetry can amplify the sensitivity to loading direction and promote coupled damage and energy-dissipation processes that differ from those of symmetric members. Experimental evidence for such members remains limited. The most relevant published study is that of Xiang et al. [33], who investigated laterally impacted SRC columns with a T-shaped steel section. Even so, the coupled effects of impact velocity (v), axial compression ratio (n), and transverse detailing, represented here by stirrup spacing in the non-densified region (s), on the full force-displacement response, residual deformation, and energy-dissipation efficiency have not been systematically quantified for L-shaped SRC columns. It also remains unclear whether the trends observed for T-shaped configurations apply to L-shaped SRC columns.

Accordingly, systematic experimental evidence and mechanistic understanding are still lacking for the lateral impact behavior of L-shaped SRC columns. This gap limits the reliability of current assessment and detailing decisions for asymmetric SRC members subjected to accidental impact.

This study therefore presents seven lateral drop-weight impact tests on SRC columns with built-in L-shaped steel sections. The effects of impact velocity (v), axial compression ratio (n), and stirrup spacing in the non-densified region (s) are examined in terms of failure pattern, impact force-displacement response, residual deformation, and energy dissipation. The main contributions are as follows:

(1) the individual and coupled effects of v, n, and s are quantified for L-shaped SRC columns under lateral impact;

(2) the role of the embedded L-shaped steel section in maintaining post-damage resistance and load-path continuity is clarified; and

(3) the response trends identify a confinement-related balance between resistance, deformation control, and energy dissipation, with implications for the preliminary assessment and transverse detailing of asymmetric SRC columns.

## 2. Materials and Methods

### 2.1. Specimen Design and Preparation

To examine the effects of impact velocity (v), axial compression ratio (n), and stirrup spacing in the non-densified region (s) on the impact performance of asymmetric steel-reinforced concrete (SRC) columns, seven specimens with an embedded L-shaped steel section were designed and fabricated in accordance with JGJ 138-2017 [34] and YB 9082-2006 [35].

A 1:2 geometric scale was adopted. The prototype was a frame column with a 600 mm × 600 mm cross-section and a height of 4.0 m, whereas the test specimens measured 300 mm × 300 mm × 2000 mm, giving a shear span ratio of approximately 3.3. This scale was selected primarily to satisfy the capacity and geometric constraints of the available drop-weight impact facility while preserving the sectional form and the main comparative parameters of the prototype member. For impact-loaded members, however, scale effects may influence inertia distribution, local stress concentration, crack propagation, and damage localization. The present results should therefore be interpreted mainly in a comparative sense within the investigated range, rather than as direct quantitative predictions for full-scale members.

**Figure 1:**
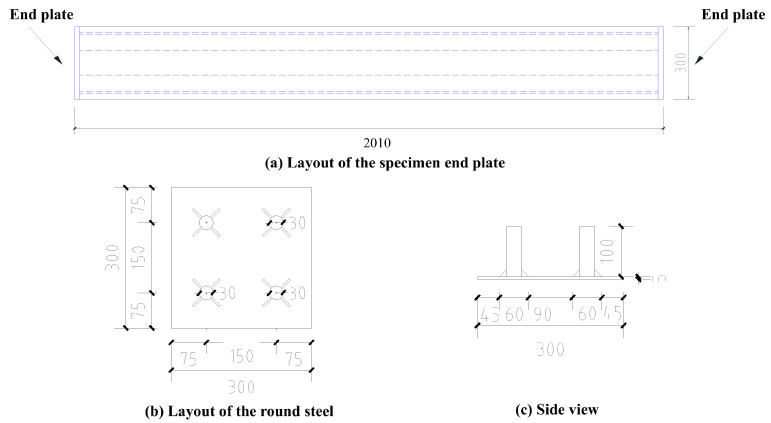
Details of the specimen end plate and round steel tube: (**a**) layout of the specimen end plate; (**b**) layout of the round steel tube; (**c**) side view (unit: mm).

The built-in L-shaped steel section was fabricated in the workshop by welding 6 mm-thick Q235B steel plates according to the design drawings before rebar assembly and concrete casting. The outer profile of the steel section was 240 mm × 100 mm. To promote uniform end load transfer and reduce stress concentration, perforated end plates (300 mm × 300 mm × 6 mm) were welded to both ends of the embedded steel section. Target axial compression ratios were imposed by post-tensioning to reproduce service axial-force conditions. The specimen geometry and end detailing are shown in Figure 1.

**Figure 2:**
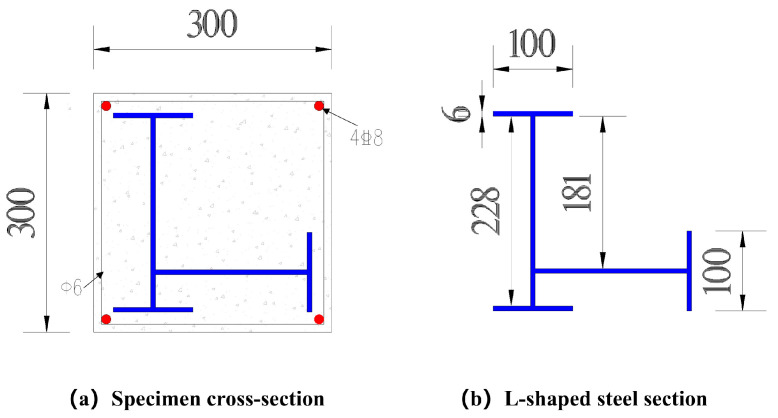
Cross-sectional details of the specimen and the built-in L-shaped steel section (unit: mm): (**a**) specimen cross-section with longitudinal reinforcement; (**b**) L-shaped steel section.

**Figure 3:**
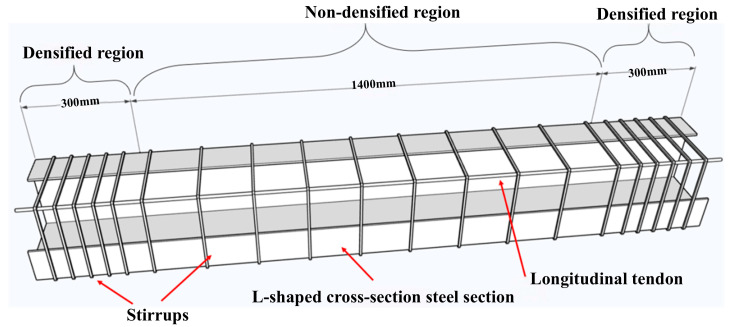
Longitudinal reinforcement layout and stirrup zoning of the specimen (unit: mm).

The longitudinal reinforcement consisted of four 8 mm-diameter HRB400 ribbed bars, and the transverse reinforcement used 6 mm-diameter HPB300 plain round bars. To enhance confinement near the supports, the stirrups were densified over a length of 300 mm at both ends, with a spacing of 50 mm. In the non-densified region, stirrup spacing s was taken as the design variable and set to 100, 150, or 200 mm. Cross-sectional details, including the reinforcement layout and the dimensions of the embedded steel section, are shown in Figure 2. The longitudinal reinforcement arrangement and stirrup zoning are presented in Figure 3.

To facilitate identification of the test parameters, the specimens were labelled using the format v–n–s, where v denotes the impact velocity (m/s), n the axial compression ratio (–), and s the stirrup spacing in the non-densified region (mm). For example, specimen v8.85–n0.1–s150 was tested at v = 8.85 m/s with n = 0.1 and s = 150 mm. The specimen matrix is summarized in Table 1. The impact velocity v was calculated from the drop height H assuming free fall.

### 2.2. Material Properties

#### 2.2.1. Concrete

All specimens were cast using ready-mixed C30 concrete. To minimize batch-related variability in the impact response, the seven columns were produced in a single casting campaign and cured under identical temperature and humidity conditions. Because the clearance around the flanges of the embedded L-shaped steel section was limited, the concrete was placed in layers and mechanically vibrated to achieve adequate compaction and to reduce the risk of honeycombing.

In parallel with specimen casting, three 150 mm concrete cubes were prepared and cured under the same conditions. At 28 days, compression tests were conducted in accordance with GB/T 50081-2019 [36]. The mean cube compressive strength was 33.7 MPa, which satisfied the target strength for the test program. The concrete mix proportions and the measured mechanical properties are summarized in Table 2.

#### 2.2.2. Steel Materials

To maintain consistency in material characterization, tensile coupons were prepared for the embedded steel section, longitudinal reinforcement, stirrups, and prestressing strands in accordance with GB/T 228.1-2010 [37]. Uniaxial tensile tests were then carried out using a universal testing machine. For each steel type, the yield strength *fy*, ultimate tensile strength *fu*, elastic modulus *Es*, and elongation at fracture *δs* were determined. The mean measured properties are listed in Table 3.

#### 2.2.3. Application of Axial Load

To examine the effect of axial force on the impact response, the axial compression ratio n was taken as a primary test variable. Following the strength superposition approach adopted in the relevant design provisions, n was defined as Equation (1), where *N* is the target axial force, *fc* is the design axial compressive strength of concrete, *Ac* is the net concrete area, *fss* is the design compressive strength of the embedded steel section, and Ass is the area of the embedded steel section.

(1) $$n=N/\left(f_{c}\cdot A_{c}+f_{ss}\cdot A_{ss}\right)$$

Considering the test objectives and the capacity of the loading system, three levels of n were adopted: 0, 0.1, and 0.2. The corresponding axial forces, calculated from Equation (1) using the measured material properties, were 0, 848.8 kN, and 1697.6 kN, respectively.

**Figure 4:**
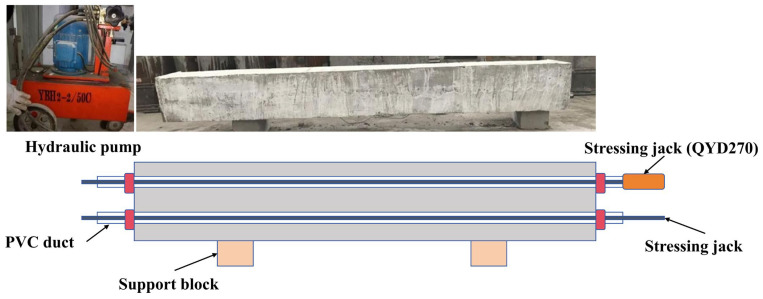
Axial load application using post-tensioning (hydraulic pump and jack, prestressing strand, anchorage, and PVC ducts).

Axial force was applied by post-tensioning. In the present study, the prestressing strand was used solely to introduce and maintain the target axial load before impact, rather than as a strengthening measure or design variable for the SRC specimens themselves. After the concrete had reached the required strength, a 1 × 7 prestressing strand with a nominal diameter of 15.2 mm (GB/T 5224-2014) [38], together with the matching anchorage, was tensioned using a hydraulic jack and pump system (Figure 4). During stressing, the applied force was controlled primarily by the oil-pressure reading and checked against the measured strand elongation. To account for anchorage seating and strand relaxation, an over-stressing–release procedure was used to stabilize the target force.

The theoretical strand elongation was estimated by Equation (2), where *Pp* is the jacking force, *L* is the effective strand length, *Ap* is the strand area, and *Ep* is the elastic modulus. The applied axial force was transferred to the specimen through the perforated end plates at both ends.

(2) $$\Delta L=\frac{P_{p}\times L}{A_{p}\times E_{p}},$$

### 2.3. Impact Test Setup and Instrumentation

#### 2.3.1. Test Setup

**Figure 5:**
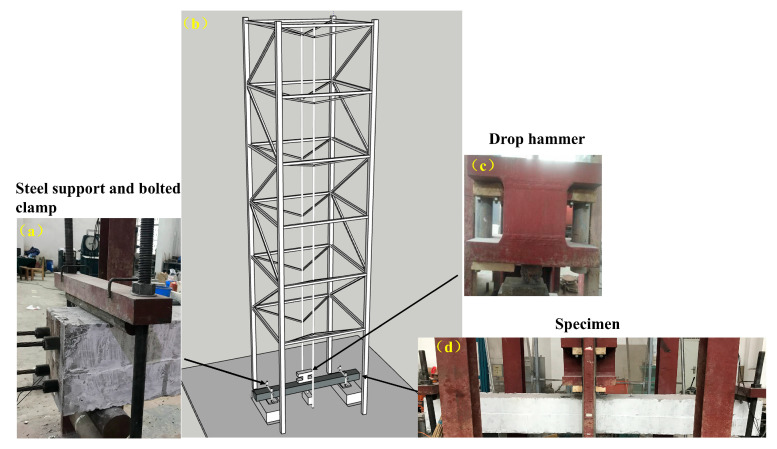
Drop-weight impact test setup and specimen arrangement: (**a**) steel support and bolted clamp (simulated pin); (**b**) overall layout; (**c**) drop hammer; (**d**) specimen.

Lateral impact tests were conducted at the Civil Engineering Laboratory of Yangtze University using a gravity-driven drop-weight system (Figure 5). The apparatus consisted of a drop hammer, vertical guide rails with a hoisting–release mechanism, a rigid steel reaction frame, and bolted supports for the specimens. The maximum drop height was 8.0 m, and the total hammer mass was 339 kg. The impact energy was controlled by varying the release height.

The striking head was made of 40Cr steel, and its deformation during impact was assumed to be negligible. A circular flat contact face with a diameter of 100 mm was adopted to maintain consistent contact conditions throughout the test program. The guide rails were lubricated before each test to reduce friction and improve repeatability.

To prevent global sliding or rotation during impact, the reaction frame and specimen supports were anchored to the strong floor using high-strength bolts. The specimens were tested under pinned–pinned boundary conditions, and the impact point was located at mid-span in all cases.

#### 2.3.2. Instrumentation and Measured Quantities

**Figure 6:**
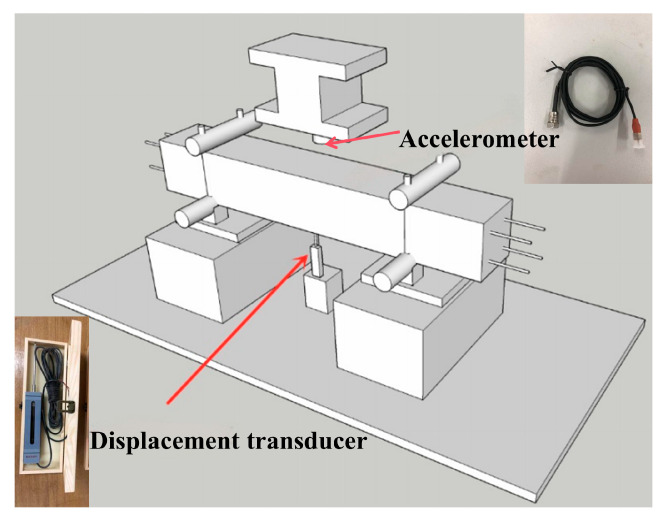
Instrumentation layout for the impact test.

The impact force history F(t) and mid-span displacement history Δ(t) were recorded for each test. The instrumentation layout is shown in Figure 6.

Impact force, F(t). Hammer acceleration was measured using an accelerometer mounted on the drop hammer. The contact force was then obtained from Newton’s second law, as given in Equation (3), where m = 339 kg is the hammer mass and a(t) is the measured acceleration time history. Force and displacement channels were recorded synchronously. Unless otherwise stated, the reported time histories are presented in their raw form, without additional filtering or smoothing.

Mid-span displacement, Δ(t). The mid-span displacement history was measured using a draw-wire displacement transducer with a bidirectional measurement range of ± 50 mm.

(3) $$F\left(t\right)=ma\left(t\right),$$

#### 2.3.3. Test Procedure

Each impact test was carried out according to the following procedure.

(1) Axial loading. After the concrete had reached the target strength, the prescribed axial force corresponding to n = 0, 0.1, or 0.2 was applied by post-tensioning. The jacking force was controlled primarily by the oil-pressure reading and checked against the measured strand elongation. The target axial force was then maintained before impact.

(2) Specimen installation and boundary conditions. The steel supports were anchored to the strong floor using high-strength bolts. The specimen was then lifted into position and connected to the end fixtures to realize pinned–pinned boundary conditions. All bolts and connectors were tightened and inspected before testing.

(3) Instrumentation. The accelerometer was mounted on the drop hammer, and the draw-wire displacement transducer was installed at the specimen mid-span, as shown in Figure 6. The acquisition channels were checked before loading to ensure stable signal output.

(4) Impact loading. The hammer was raised to the prescribed drop height corresponding to the target impact velocity and then released under free-fall conditions. The hammer impacted the specimen at mid-span, and all measurement channels were triggered simultaneously to record the impact event.

(5) Post-impact inspection. After the test, the specimen was unloaded and visually inspected. Crack development, local crushing, spalling, and residual deformation were recorded for subsequent analysis.

## 3. Experimental Results and Analysis

### 3.1. Failure Modes and Damage Patterns

**Figure 7:**
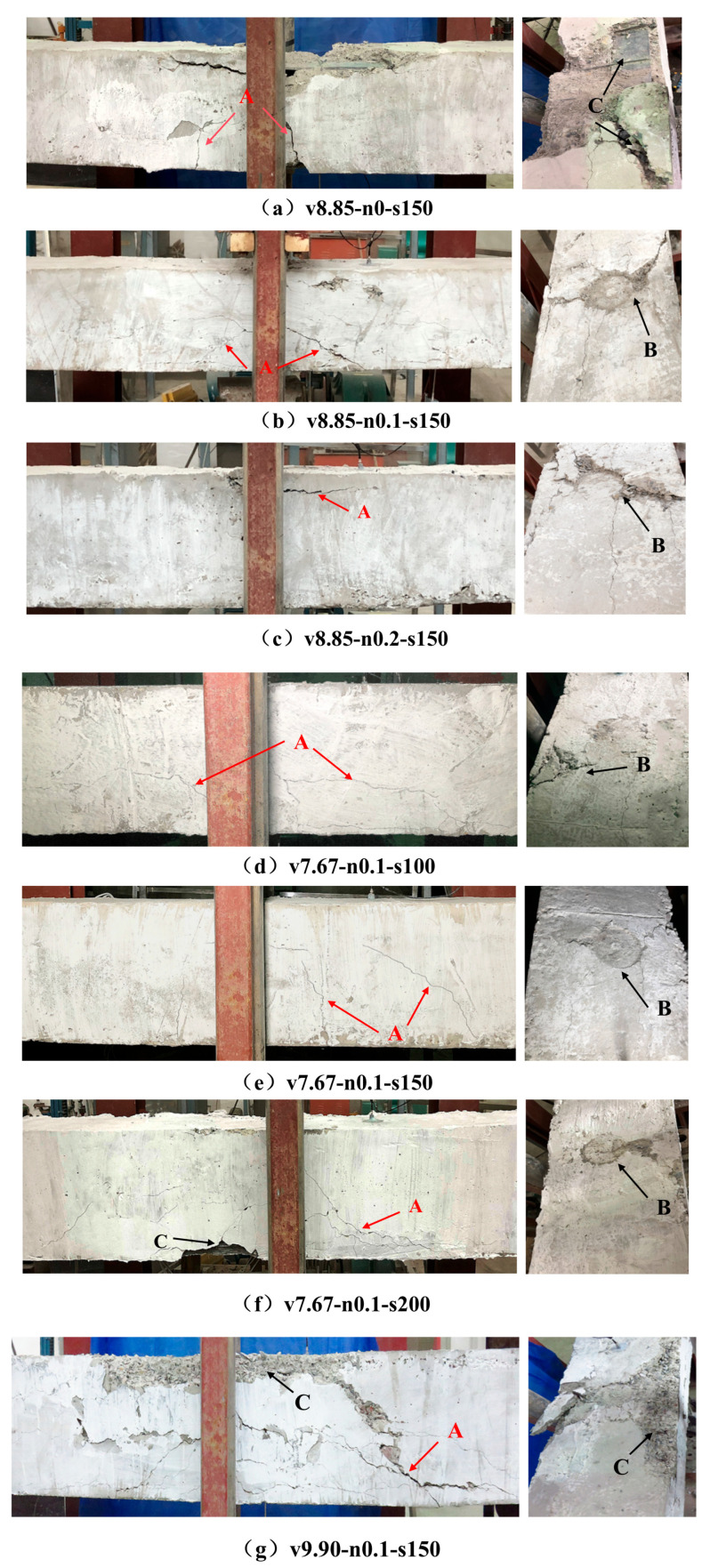
Typical post-impact damage patterns of L-shaped SRC column specimens: (**a**) v8.85–n0–s150; (**b**) v8.85–n0.1–s150; (**c**) v8.85–n0.2–s150; (**d**) v7.67–n0.1–s100; (**e**) v7.67–n0.1–s150; (**f**) v7.67–n0.1–s200; and (**g**) v9.90–n0.1–s150. Letter markers indicate representative damage features, where A denotes cracking, B denotes impact dent or local crushing, and C denotes concrete spalling or local exposure of the embedded steel section. The impact contact area, corresponding to the hammer head, had a nominal diameter of 100 mm, which provides a dimensional reference for the scale of the observed local damage.

This section describes the macroscopic damage observed in the L-shaped SRC columns after lateral drop-weight impact. All specimens remained stable after impact, and no global instability or collapse was observed. Damage was consistently concentrated in the mid-span impact region and its vicinity (Figure 7). Overall, the response was governed by a flexure–shear-dominated failure pattern, characterized by diagonal cracking, local crushing beneath the impact point, and concrete spalling.

Diagonal cracks initiated near the impact zone and propagated toward both supports, forming the dominant crack pattern on the side faces. As damage developed, vertical flexural cracks also appeared in the tensile region. On the impacted face, local crushing produced a near-circular impact dent whose size was comparable to that of the flat striking head (approximately 100 mm). Under the highest impact velocity, represented by specimen v9.90–n0.1–s150, concrete spalling became more extensive and the embedded L-shaped steel was locally exposed. Longitudinal openings were also observed along the steel–concrete interface near the impact region, indicating localized separation and degradation of composite action.

No global buckling of the embedded L-shaped steel section was observed in any specimen, and the stirrup cage remained intact. These observations indicate that the internal steel skeleton, together with transverse confinement, maintained a continuous internal load path and preserved post-impact integrity.

Clear trends were observed within each single-parameter series. Increasing the axial compression ratio reduced crack opening and mitigated spalling near the impact region. Increasing stirrup spacing weakened transverse confinement, allowing diagonal cracks to develop more fully and enlarging the damaged zone. Impact velocity had the most pronounced effect, leading to a deeper impact dent, more severe spalling, and wider crack propagation. In summary, L-shaped SRC columns exhibited a flexure–shear-dominated damage pattern under lateral impact. Impact velocity governed the overall damage severity, whereas axial compression ratio and stirrup spacing mainly influenced crack development and local concrete deterioration. The embedded L-shaped steel section played a key role in preserving overall integrity after impact.

### 3.2. Impact Response Indices

To quantify the lateral impact response of the L-shaped SRC columns, the impact force and mid-span displacement time histories were recorded synchronously in each test. Based on these measurements, the main response indices used for subsequent comparison were the peak impact force Fmax, the average plateau force Fave, the impact duration T, the maximum mid-span displacement Δmax, and the residual mid-span displacement Δres. Here, Fave is defined as the mean force over the quasi-stable plateau segment of the force–time history, and T is taken as the time interval from the onset of rapid force rise to the point at which the force decays to approximately zero. The absorbed energy E_ab_ was calculated from the measured force–displacement (F–Δ) curve, and the energy absorption ratio was evaluated as η = E_ab_/E, where E is the nominal input impact energy. Table 4 summarizes the applied axial force N, the nominal impact energy E, and the measured response indices for all specimens.

#### 3.2.1. Impact Force–Time Histories

**Figure 8:**
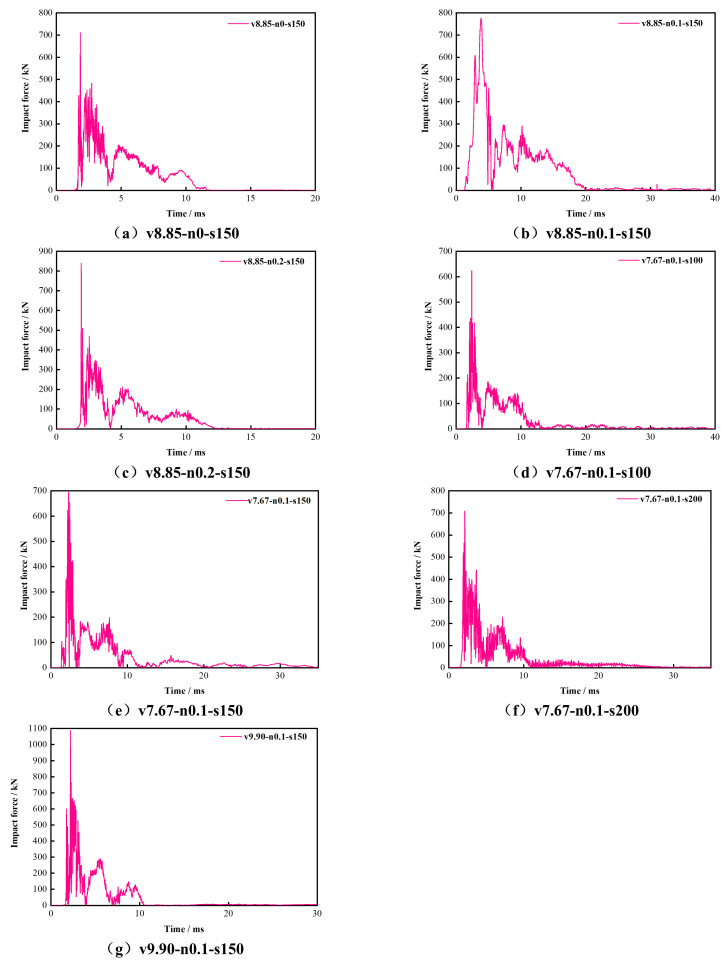
Impact force–time histories of the specimens.

**Figure 9:**
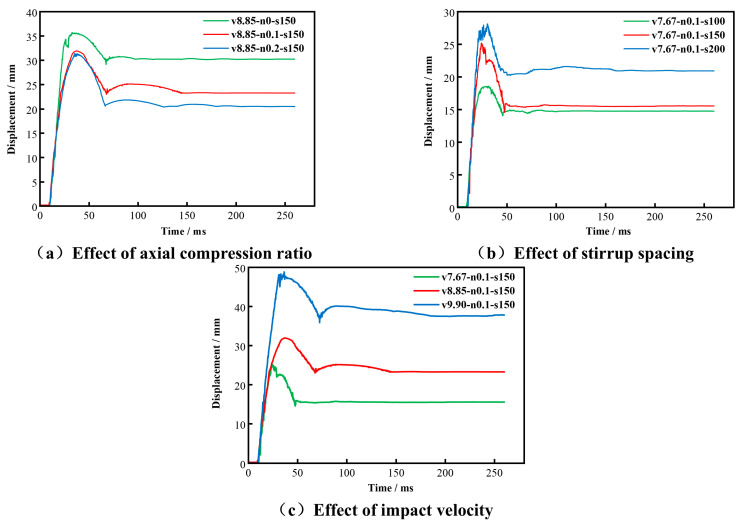
Mid-span displacement–time histories of specimens with different (**a**) axial compression ratios, (**b**) stirrup spacings, and (**c**) impact velocities.

**Figure 10:**
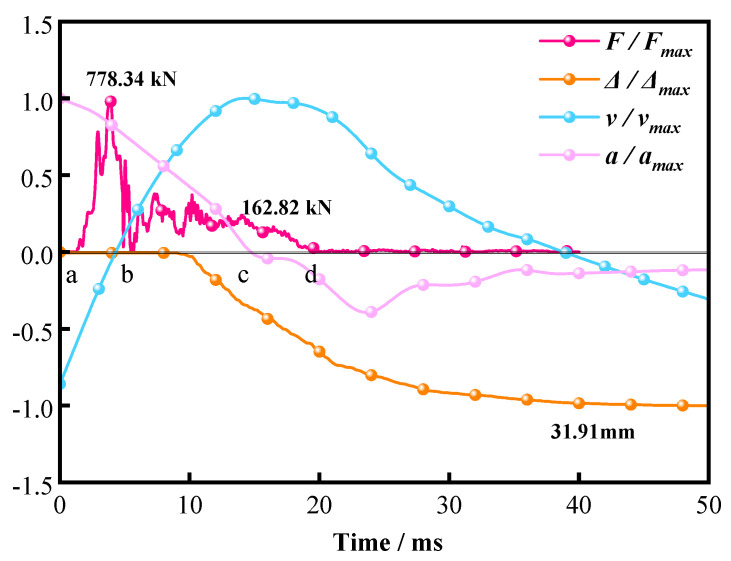
Normalized time histories of impact force F(t), mid−span displacement Δ(t), mid-span velocity v(t), and mid-span acceleration a(t) for representative specimen v8.85–n0.1–s150.

As shown in Figure 8, the impact force–time histories of all specimens exhibited a consistent peak–plateau–decay pattern. Immediately after contact, the force rose sharply to Fmax, then fluctuated around a quasi-stable level represented by Fave, and finally decayed to near zero as the impact event ended. Figure 9 shows the corresponding displacement–time histories, while Figure 10 illustrates the stage decomposition for a representative specimen. In the following analyses, Fmax, Fave, and T are used to characterize the amplitude, sustained resistance, and duration of the impact response.

#### 3.2.2. Displacement–Time Histories

The mid-span displacement–time histories are shown in Figure 9. For all specimens, the displacement increased rapidly after impact, followed by rebound and damped oscillation, and eventually approached a stable residual value. No specimen exhibited sustained displacement divergence or a secondary growth branch, indicating that global instability did not occur and that overall member integrity was retained after impact. The corresponding stage decomposition is also illustrated in Figure 10. In the subsequent analysis, Δmax and Δres are used to represent the peak deformation demand and the retained irreversible deformation, respectively.

#### 3.2.3. Representative Stage Decomposition of the Impact Process

To illustrate the typical impact response of L-shaped SRC columns, specimen v8.85–n0.1–s150 was selected as a representative case. Figure 10 shows the normalized histories of impact force F(t), mid-span displacement Δ(t), and the corresponding mid-span velocity v(t) and acceleration a(t) derived from Δ(t). To reduce noise amplification during numerical differentiation, v(t) and a(t) were obtained from a smoothed displacement record. The downward direction is taken as positive. Four characteristic instants (a–d) are marked to indicate the main response states.

As shown in Figure 10, the impact process may be divided into three successive stages. From a to b, the response is dominated by contact establishment and rapid force growth. From b to c, the force fluctuates around a quasi-stable plateau while the displacement continues to increase to its maximum value, representing the principal deformation phase. From c to d, the specimen undergoes unloading and rebound, and the response gradually decays toward a stable residual state. This stage decomposition is used only to assist interpretation of the measured time histories; the quantitative comparisons in the following sections are based on the response indices listed in Table 4.

### 3.3. Energy Absorption Characteristics

The impact resistance of the L-shaped SRC columns was further evaluated from an energy perspective using the nominal input energy E, the absorbed energy E_ab_, and the energy absorption ratio η. The nominal input energy at the instant of hammer–specimen contact was calculated from the hammer mass and impact velocity, as given in Equation (4), where *m* is the hammer mass and *v* is the impact velocity determined from the drop height assuming free fall. The absorbed energy *E_ab_* was obtained by integrating the measured force–displacement (F–Δ) curve, as given in Equation (5). The energy absorption ratio was then defined as η = E_ab_/E, as expressed in Equation (6).

(4) $$E=\frac{1}{2}mv^{2},$$

(5) $$E_{ab}=\int_{0}^{\Delta_{\max}}Fd\Delta ,$$

(6) $$\eta =\frac{E_{ab}}{E}\times 100\%,$$

**Figure 11:**
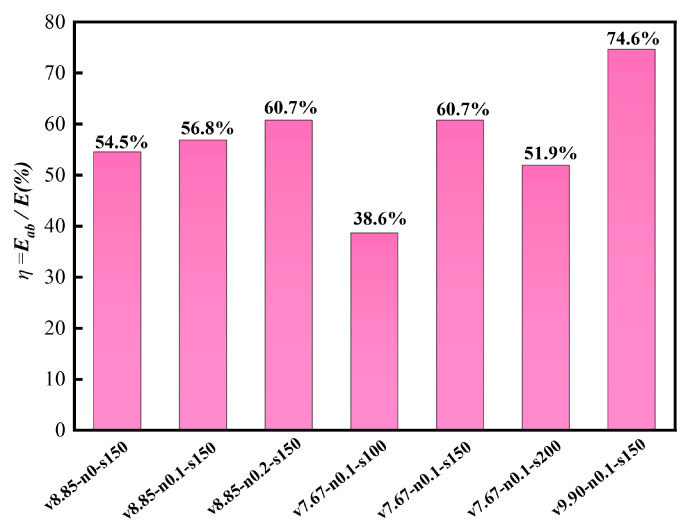
Energy absorption ratio, η (=E_ab_/E), of the tested specimens.

The calculated values of η are listed in Table 4. For all specimens, η ranged from 38.6% to 74.6%, indicating that the tested L-shaped SRC columns were able to dissipate a substantial portion of the input impact energy. For clarity, only the energy absorption ratio η is compared graphically in Figure 11, whereas the detailed absorbed energy values are given in Table 4.

Within the investigated parameter range, impact velocity had the most pronounced influence on energy absorption efficiency. For the reference stirrup spacing (s = 150 mm) and axial compression ratio (n = 0.1), increasing the impact velocity from 7.67 m/s to 9.90 m/s increased η from 60.7% to 74.6%. By contrast, increasing the axial compression ratio from 0 to 0.2 under v = 8.85 m/s and s = 150 mm increased η more moderately, from 54.5% to 60.7%. The effect of stirrup spacing was non-monotonic: under v = 7.67 m/s and n = 0.1, the specimen with s = 150 mm exhibited the highest η (60.7%), whereas the specimen with s = 100 mm showed a much lower value (38.6%).

Overall, the energy-based results are consistent with the force–displacement and damage observations presented above. Figure 11 therefore provides a compact comparison of the energy absorption efficiency of the tested specimens, while the corresponding absorbed energy values are reported in Table 4.

### 3.4. Parametric Analysis

#### 3.4.1. Effect of Axial Compression Ratio (n)

To evaluate the influence of axial compression ratio on the impact response, three specimens with n = 0, 0.1, and 0.2 were compared. These specimens, namely v8.85–n0–s150, v8.85–n0.1–s150, and v8.85–n0.2–s150, were tested under the same impact velocity (v = 8.85 m/s) and stirrup spacing (s = 150 mm).

**Figure 12:**
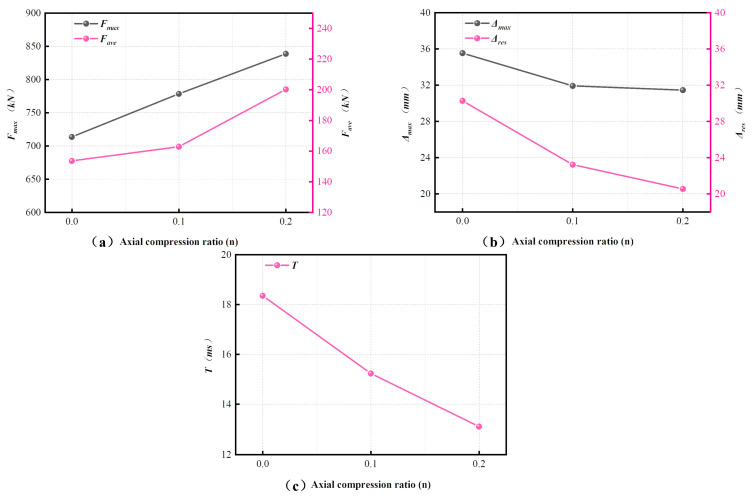
Effect of axial compression ratio n on (**a**) Fmax and Fave, (**b**) Δmax and Δres, and (**c**) impact duration T.

As n increased from 0 to 0.2, the overall resistance of the specimens increased (Figure 12a). The peak impact force Fmax rose from 713.49 kN to 838.62 kN, corresponding to an increase of 17.5%, while the average plateau force Fave increased from 153.54 kN to 200.19 kN, or by 30.4%. At the same time, the impact duration T decreased from 18.35 ms to 13.10 ms (Figure 12c), representing a reduction of 28.6%.

The deformation response showed the opposite trend (Figure 12b). As n increased, both the maximum mid-span displacement Δmax and the residual displacement Δres decreased. Δmax decreased from 35.52 mm to 31.44 mm (11.5%), whereas Δres decreased from 30.26 mm to 20.54 mm (32.1%). These observations are consistent with the damage patterns described in Section 3.1, where increasing axial compression ratio was associated with less extensive cracking and less severe concrete spalling.

Overall, within the investigated range (n ≤ 0.2), axial compression improved impact resistance and deformation control. Increasing n led to higher Fmax and Fave, but lower Δmax, Δres, and T, indicating a more compact impact response with reduced residual deformation.

#### 3.4.2. Effect of Impact Velocity (v)

To evaluate the influence of impact velocity on the dynamic response, three specimens with v = 7.67, 8.85, and 9.90 m/s were compared. These specimens, namely v7.67–n0.1–s150, v8.85–n0.1–s150, and v9.90–n0.1–s150, were tested under the same axial compression ratio (n = 0.1) and stirrup spacing (s = 150 mm). The corresponding response indices are summarized in Table 4.

**Figure 13:**
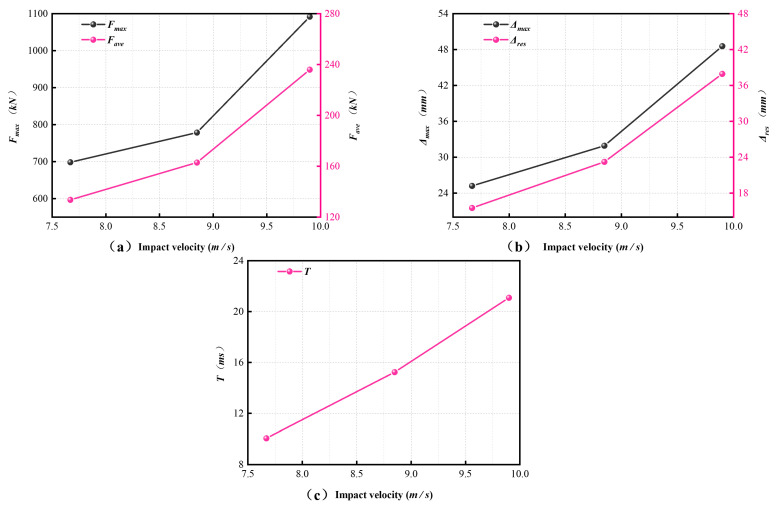
Effect of impact velocity v on (**a**) Fmax and Fave, (**b**) Δmax and Δres, and (**c**) impact duration T.

Both the peak impact force Fmax and the average plateau force Fave increased markedly with increasing v (Figure 13a). As v increased from 7.67 m/s to 9.90 m/s, Fmax increased from 698.02 kN to 1091.37 kN, corresponding to an increase of 56.3%, while Fave increased from 133.51 kN to 235.84 kN, or by 76.6%. The impact duration T increased from 10.04 ms to 21.08 ms (Figure 13c), representing an increase of 110.0%.

The displacement response was highly sensitive to impact velocity (Figure 13b). The maximum mid-span displacement Δmax increased from 25.20 mm to 48.54 mm (92.6%), while the residual displacement Δres increased from 15.52 mm to 37.92 mm (144.3%). These trends are consistent with the damage patterns described in Section 3.1, where higher impact velocities were associated with deeper impact dents, more severe concrete spalling, and wider crack propagation.

Overall, within the investigated range, impact velocity had the strongest influence on the amplitude and severity of the dynamic response. Increasing v led to higher Fmax and Fave, longer impact duration, and larger peak and residual displacements.

#### 3.4.3. Effect of Stirrup Spacing (s)

**Figure 14:**
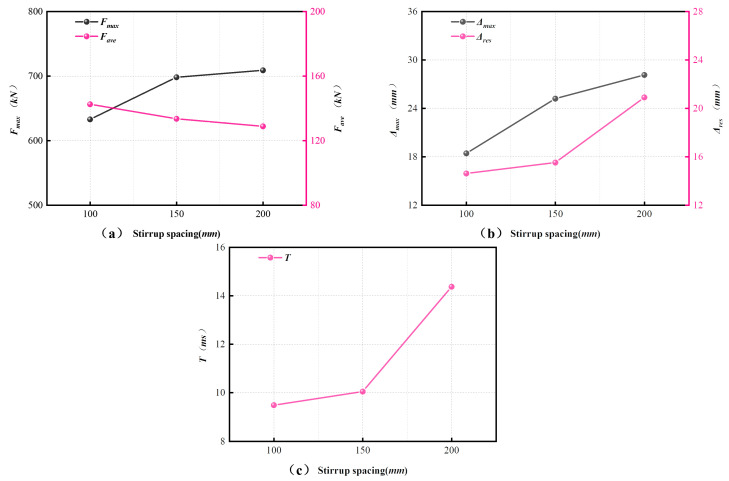
Effect of stirrup spacing s on (**a**) Fmax and Fave, (**b**) Δmax and Δres, and (**c**) impact duration T.

Stirrup spacing in the non-densified region affects the transverse confinement of the core concrete and, in turn, the impact response of the SRC columns. To examine this effect, three specimens with s = 100, 150, and 200 mm were compared under the same impact velocity (v = 7.67 m/s) and axial compression ratio (n = 0.1). The corresponding response indices are listed in Table 4 and shown in Figure 14.

As shown in Figure 14a, the peak impact force Fmax increased from 632.86 kN to 708.85 kN as s increased from 100 mm to 200 mm, corresponding to an increase of 12.0%. By contrast, the average plateau force Fave remained within a relatively narrow range (128.83–142.51 kN) and showed a slight overall decrease with increasing s. The displacement response exhibited greater sensitivity to stirrup spacing (Figure 14b). Δmax increased from 18.42 mm to 28.13 mm (52.7%), while Δres increased from 14.62 mm to 20.90 mm (42.9%).

The impact duration T increased from 9.48 ms to 14.37 ms (51.6%) as s increased (Figure 14c). These results indicate that weaker transverse confinement in the non-densified region was associated with larger deformation demand and a longer deformation process under impact.

Taken together, the effect of stirrup spacing was non-monotonic. Increasing s produced only a modest increase in Fmax, but led to larger displacement demand and longer impact duration. The corresponding variation in energy absorption efficiency is presented separately in Section 3.3.

## 4. Discussion

### 4.1. Effects of Axial Compression Ratio on Dynamic Response and Failure Modes

Within the investigated range (n ≤ 0.2), the axial compression ratio had a pronounced effect on the lateral impact response of the L-shaped SRC columns. The main consequences were higher resistance, lower deformation demand, and more localized damage.

When n increased from 0 to 0.2, the resistance capacity increased substantially. The peak impact force $\left(Fmax\right)$ rose from 713.49 kN to 838.62 kN (17.5%), and the average plateau force $\left(Fave\right)$ increased from 153.54 kN to 200.19 kN (30.4%). By contrast, the deformation response was reduced. The maximum mid-span displacement $\left(\Delta max\right)$ decreased from 35.52 mm to 31.44 mm (11.5%), while the residual displacement $\left(\Delta res\right)$ decreased more markedly from 30.26 mm to 20.54 mm (32.1%). The impact duration (T) also shortened from 18.35 ms to 13.10 ms (28.6%). These changes are consistent with the damage observations in Section 3.1, where higher axial compression ratios suppressed crack propagation and reduced concrete spalling.

This behavior is broadly consistent with earlier impact studies on RC and SRC columns [10,11,33], which likewise showed that moderate axial compression can enhance impact resistance while limiting lateral deformation. A more detailed section-type comparison with previously reported T-shaped and cruciform SRC columns is given in Section 4.6.

Mechanically, axial pre-compression enhances section-level confinement and stabilizes force transfer between steel and concrete, allowing resistance to be mobilized earlier in the impact event. It also restrains diagonal flexural–shear cracking, thereby confining damage more closely to the impact region and the steel–concrete interface. The accompanying increase in energy absorption ratio further indicates improved internal coordination between the steel skeleton and the surrounding concrete.

This beneficial effect should, however, be interpreted only within the investigated range. With increasing lateral displacement, the applied axial load can magnify the impact-induced bending moment through P–Δ effects, thereby reducing the residual stability margin. Comparable stiffness- and stability-related behaviour has been reported in analytical and numerical studies of slender concrete columns, in which flexural rigidity controls deformation growth and the onset of instability under combined axial and lateral actions [39]. Although those studies were not performed under impact loading, they still help contextualize the present observations. Moreover, under severe impact, pronounced local crushing and concrete spalling were observed, indicating that any beneficial effect of axial compression must be evaluated together with the stability constraints that become increasingly significant at larger lateral deformations.

### 4.2. Dominant Role of Impact Velocity

Impact velocity was the dominant parameter governing the lateral impact response of the present L-shaped SRC columns, primarily because it directly determined the kinetic energy input of the drop hammer. Within the investigated range, increasing v led to clear and systematic increases in force response, deformation demand, and impact duration.

As v increased from 7.67 m/s to 9.90 m/s, the peak impact force (Fmax) increased by 56.3%, and the average plateau force (Fave) increased by 76.6%. The impact duration (T) increased by 110.0%. The corresponding deformation response was even more sensitive: the maximum mid-span displacement (Δmax) increased by 92.6%, while the residual displacement (Δres) increased by 144.3%. These results are consistent with the damage observations in Section 3.1, where higher impact velocities produced deeper local crushing, more extensive concrete spalling, and wider crack propagation.

These trends are broadly consistent with previous studies on RC, SRC, and steel–concrete composite members under lateral impact [9,10,11,30,33]. A more detailed quantitative comparison with previously reported T-shaped and cruciform SRC columns is presented in Section 4.6.

In addition to the increase in impact energy, strain-rate effects in concrete and steel may also have contributed to the response at higher impact velocities. Under impact loading, rate-dependent increases in apparent material strength and stiffness may influence peak force development, local crushing, and energy dissipation. Since local strain rates were not measured directly in the present tests, this effect cannot be quantified separately and is therefore discussed here only as a plausible physical contribution rather than as a calibrated rate-dependent mechanism.

Despite the more severe local damage observed at higher impact velocities, the energy absorption ratio (η) also increased. For specimen v9.90–n0.1–s150, η reached 74.6%, while the absorbed energy (E_ab_) increased from 6.06 kJ to 12.38 kJ as v rose from 7.67 m/s to 9.90 m/s. Post-test observations further showed that even when concrete crushing and spalling exposed part of the embedded steel, the built-in L-shaped steel section did not exhibit global buckling, and the specimens retained overall stability. This suggests that the internal steel skeleton continued to provide load-path continuity after local concrete degradation, thereby enabling substantial energy dissipation even under high-velocity impact.

Overall, within the investigated range, impact velocity was the most influential parameter controlling response severity under lateral impact.

### 4.3. Influence of Stirrup Spacing on Deformation and Energy Dissipation

Stirrup spacing s had a clear influence on the impact response of the L-shaped SRC columns because it governed the level of transverse confinement in the non-densified region. Within the investigated range, this effect was distinctly non-monotonic. The main consequences were a modest variation in peak resistance, a stronger sensitivity of deformation demand, and a non-monotonic change in energy absorption efficiency.

As s increased from 100 mm to 200 mm, the peak impact force (Fmax) increased from 632.86 kN to 708.85 kN, corresponding to a rise of 12.0%. By contrast, the average plateau force (Fave) remained within a relatively narrow range but decreased slightly overall, from 142.51 kN at s = 100 mm to 128.83 kN at s = 200 mm. This difference indicates that stirrup spacing had a limited effect on the instantaneous peak response but a more noticeable influence on the sustained resistance developed during the impact process.

The deformation response was considerably more sensitive to stirrup spacing. When s increased from 100 mm to 200 mm, the maximum displacement (Δmax) increased from 18.42 mm to 28.13 mm (52.7%), while the residual displacement (Δres) increased from 14.62 mm to 20.90 mm (42.9%). These changes are consistent with the damage observations in Section 3.1, where wider stirrup spacing was associated with more extensive crack development and a larger damaged zone.

The variation in energy absorption ratio (η) was also non-monotonic. The highest value (60.7%) was recorded at s = 150 mm, whereas the specimen with the smallest spacing (s = 100 mm) showed a substantially lower value (38.6%). The specimen with the widest spacing (s = 200 mm) gave an intermediate value (51.9%), confirming that neither the densest nor the widest transverse reinforcement arrangement produced the most favorable overall response. Instead, the intermediate spacing provided a better balance among resistance, deformation control, and energy dissipation within the tested range.

A broadly similar role of transverse confinement has been reported in earlier impact studies on RC and SRC columns [9,33]. Reduced stirrup spacing generally improves crack control and preserves member integrity, whereas larger spacing leads to greater deformation demand and more extensive damage. A more detailed quantitative comparison with previously reported T-shaped and cruciform SRC columns is presented in Section 4.6.

Within the investigated range, s = 150 mm provided the most balanced overall response among the tested cases, rather than a definitive optimum. The corresponding normalized trend of η is discussed further in Section 4.4.

### 4.4. Quantification of Parametric Trends and Strain-Rate-Related Interpretation

To move beyond purely descriptive comparison, the effects of the three governing parameters were further expressed in normalized form using three key response indices, namely the peak impact force Fmax, the maximum displacement Δmax, and the energy absorption ratio η. For the impact-velocity and axial-compression-ratio groups, specimen v8.85–n0.1–s150 was taken as the reference. For the stirrup-spacing group, specimen v7.67–n0.1–s150 was used as the reference, since this group was tested under a constant impact velocity (v = 7.67 m/s) and axial compression ratio (n = 0.1). The estimated response may be written as

(7) $$R=R_{ref}\cdot \phi_{x}\left(x\right),$$

where *R* is the response quantity, *R_ref_* is the corresponding value of the reference specimen, and *ϕ_x_*(*x*) is a normalized correction function associated with the varying parameter x (v, n, or s). In the present study, *ϕ_x_*(*x*) was represented by low-order fitting within each controlled comparison group, and the corresponding fitted expressions are summarized in Appendix A.

**Figure 15:**
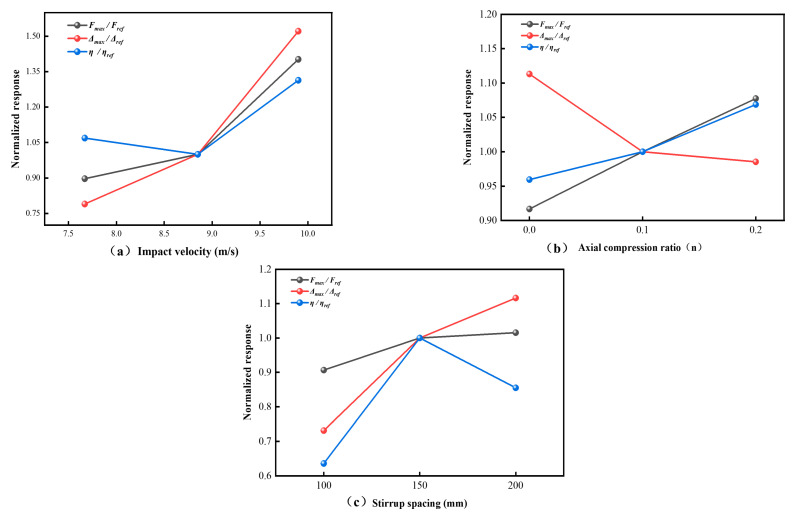
Normalized trends of Fmax, Δmax, and η under different parameter variations: (**a**) impact velocity for specimens with n = 0.1 and s = 150 mm, (**b**) axial compression ratio for specimens with v = 8.85 m/s and s = 150 mm, and (**c**) stirrup spacing for specimens with v = 7.67 m/s and n = 0.1.

Figure 15 summarizes the resulting normalized trends for Fmax, Δmax, and η under different parameter variations. Increasing impact velocity led to clear increases in normalized Fmax and normalized Δmax. Over the tested velocity range, Δmax increased by 92.6%, compared with 56.3% for Fmax, indicating that deformation demand was more sensitive to impact velocity than peak resistance. The normalized η values likewise reached their highest level at the largest impact velocity. Among the three indices, Δmax showed the strongest sensitivity to impact velocity.

The influence of impact velocity should also be interpreted in relation to strain-rate sensitivity. A characteristic member-level strain-rate scale may be estimated as $\dot{\varepsilon }∼v/L$, giving an order of magnitude of approximately 4–5 s^−1^ for the present tests based on v=7.67–9.90 m/s and L=2.0 m. This estimate is intended only to indicate the overall loading-rate level of the member, rather than a directly measured local material strain rate. Within this range, established dynamic increase factor (DIF) models suggest that both concrete and steel may exhibit non-negligible rate-dependent strength enhancement under impact loading [40,41,42]. Similar lateral-impact studies on SRC columns have also reported peak strain-rate levels on the order of 10–15 s^−1^ in reinforcing steel and embedded steel sections under comparable impact conditions [43]. This local level exceeds the present member-scale estimate, which is reasonable because strain-rate concentration is expected in the plastic hinge region. The observed velocity effect should therefore be understood as the combined result of increasing input energy and rate-related material enhancement, rather than as a pure energy-input effect alone. Since local strain rates were not measured directly, this interpretation should be regarded as approximate.

Increasing axial compression ratio was associated with a moderate increase in normalized Fmax and η, while normalized Δmax decreased slightly. By contrast, the effect of stirrup spacing was non-monotonic. To describe this feature more explicitly, the normalized energy absorption ratio in the stirrup-spacing group was approximated by the following quadratic relation:

(8) $$\frac{\eta }{\eta_{ref}}=1+0.1096X-0.2545X^{2},X=\frac{s-150}{50},$$

where *X* = 0 corresponds to *s* = 150 mm. This relation captures the non-monotonic trend observed at the three tested spacing levels within the investigated range. The corresponding fitted expressions for normalized Fmax and Δmax, together with those for the other parameter groups, are summarized in Appendix A.

These normalized results further indicate that stirrup spacing governs a trade-off among resistance, deformation control, and energy dissipation. Within the investigated range, s = 150 mm provided the most balanced overall response among the tested cases rather than a definitive optimum. Overall, the present normalization framework provides a quantitative basis for comparing the relative influence of the three governing parameters and for supporting preliminary within-range interpretation. It should not, however, be treated as a standalone predictive model for design, particularly in view of the limited specimen matrix and the absence of repeated tests.

### 4.5. Simplified Mechanical Interpretation of Impact Resistance and Energy Dissipation

**Figure 16:**
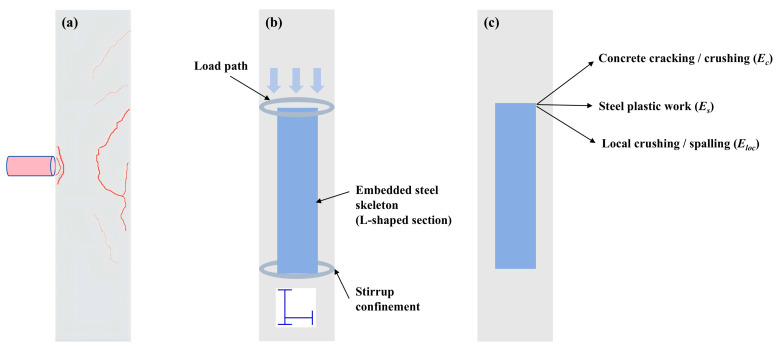
Conceptual illustration of the simplified mechanical interpretation of lateral impact resistance in L-shaped SRC columns: (**a**) impact damage pattern; (**b**) retained load path and confinement mechanism; (**c**) principal energy dissipation pathways.

To clarify the observed response, the lateral impact behavior of the present L-shaped SRC columns is interpreted through a simplified force-transfer and energy-dissipation framework. At the sectional level, the residual resistance may be conceptually expressed as

(9) $$M_{res}\approx M_{c}+M_{rs}+M_{ss},$$

where *M_c_*, *M_rs_*, and *M_ss_* denote the respective contributions of the concrete, the reinforcing steel, and the embedded steel section to the coupled flexural-shear resistance of the section. Equation (9) is introduced only to distinguish the relative roles of the constituent components and is not intended as a predictive capacity model. At the onset of impact, these components act together to resist the imposed demand. As cracking, crushing, and local spalling progressively develop in the surrounding concrete, the concrete contribution diminishes. By contrast, the embedded steel section and the longitudinal reinforcement continue to preserve the internal load path. This retained load path helps explain why severe local damage did not trigger global collapse in the present tests. The conceptual basis of this interpretation is illustrated in Figure 16, in which the damage pattern and retained load path are shown in Figure 16a,b, while the principal energy dissipation pathways are summarized in Figure 16c.

The effect of stirrup spacing may also be interpreted in terms of confinement efficiency. In simplified form, the confinement level may be represented by the transverse reinforcement index

(10) $$I_{conf}=\rho_{sv}\frac{f_{yv}}{f_{c}}$$

where ρ_sv_ is the volumetric stirrup ratio, *f_yv_* is the stirrup yield strength, and *f_c_* is the concrete compressive strength. This index is used here only to indicate the relative confinement level provided by the stirrups, rather than as a calibrated design parameter. Since $\rho_{sv}\propto 1/s$, the confinement efficiency decreases as the stirrup spacing s increases. Reduced confinement promotes crack localization, accelerates deterioration of the concrete core, and increases residual deformation. This interpretation is consistent with the displacement response observed in the present tests and also helps explain the non-monotonic variation in energy absorption efficiency discussed in Section 4.4. Within the investigated range, the intermediate spacing level (s = 150 mm) provided the most balanced overall response among the tested cases rather than representing a definitive optimum.

As defined in Equation (5), the absorbed energy *E_ab_* may be interpreted in terms of three main dissipation pathways: energy dissipated through concrete cracking and crushing (*E_c_*), plastic work in the reinforcing bars and the embedded steel section (*E_s_*), and local contact-related dissipation near the impact zone (*E_loc_*), including local crushing and spalling. In the present specimens, concrete mainly provided local bearing and confinement during the initial stage of impact. As concrete damage accumulated, the steel skeleton carried an increasing share of the post-damage resistance. The overall response may therefore be understood as a progressive shift in both load-carrying responsibility and energy dissipation, from a concrete-dominated stage to a steel-sustained stage.

This treatment remains a simplified mechanical interpretation rather than a calibrated analytical model. Even so, it provides a clearer basis for discussing load-path continuity, confinement efficiency, and the main energy dissipation pathways in L-shaped SRC columns under lateral impact.

### 4.6. Quantitative Benchmarking Against Previously Reported SRC Impact Tests

To place the present results in a broader context, a quantitative benchmarking analysis was conducted against previously reported lateral impact tests on T-shaped and cruciform SRC columns. Across the three studies, the specimens were designed as 1:2 scaled SRC columns with a 300 × 300 mm section and a member length of 2000 mm, and the main variables were defined in terms of impact velocity, axial compression ratio, and stirrup spacing. This provides a consistent basis for comparing the influence of section type. Table 5 summarizes the sensitivity of the main response indices under matched loading conditions.

Under the matched velocity case (v = 7.67 → 9.90 m/s, n = 0.1, s = 150 mm), the increase in maximum displacement was 92.6% for the L-shaped columns, compared with 80.2% for the T-shaped columns and 88.0% for the cruciform columns. More notably, the increase in residual-like displacement was 144.3% for the L-shaped columns, versus 107.3% and 94.7% for the T-shaped and cruciform columns, respectively. This indicates that the L-shaped section was more sensitive to irreversible deformation accumulation as impact severity increased. The corresponding increase in peak force was 56.4% for the L-shaped columns, 32.2% for the T-shaped columns, and 56.1% for the cruciform columns.

Under the matched axial-loading case (n = 0 → 0.2, v = 8.85 m/s, s = 150 mm), the reduction in residual-like displacement was 32.1% for the L-shaped columns, compared with 17.5% for the T-shaped columns and 9.9% for the cruciform columns. By contrast, the increase in peak impact force was most pronounced in the T-shaped columns (+72.1%), compared with +17.5% for the L-shaped columns and +13.1% for the cruciform columns. These results suggest that the response of SRC columns under impact is strongly section-dependent, and that the L-shaped configuration is particularly sensitive in terms of deformation accumulation and confinement-related deformation control, whereas the T-shaped configuration shows a stronger axial-load effect on peak resistance.

For the stirrup-spacing case (s = 100 → 200 mm, v = 7.67 m/s, n = 0.1), the increase in maximum displacement was 52.7% for the L-shaped columns, compared with 38.5% for the T-shaped columns and 47.9% for the cruciform columns. This again indicates that the L-shaped section was more sensitive to changes in transverse confinement. In terms of energy dissipation, the present L-shaped specimens showed an energy absorption ratio of 38.6–74.6%, while the previously reported cruciform specimens showed a comparable range of 33.2–73.9%. At the matched medium-spacing case (s = 150 mm), the L-shaped specimen had η = 60.7%, compared with 49.4% for the cruciform specimen. The T-shaped study did not report absorbed energy directly, so η is not compared for that series.

Taken together, these comparisons show that the present L-shaped SRC columns do not simply reproduce the response trends reported for previously tested asymmetric or symmetric SRC sections. Their most distinctive feature is the stronger sensitivity of residual deformation to both impact severity and confinement variation, while their energy dissipation efficiency remains at least comparable to that of previously tested cruciform SRC columns. This section-type dependence should be taken into account when assessing the impact resistance and detailing strategy of asymmetric SRC members.

### 4.7. Uncertainty, Scaling Considerations, and Applicability

The present findings should be interpreted in light of the limited specimen matrix and the absence of repeated tests under identical conditions. The observed differences among specimens are sufficient to support the main response trends discussed above, particularly those associated with impact velocity, axial compression ratio, and stirrup spacing. Even so, the quantitative magnitude of some differences should still be treated with caution. Because repeated tests were not available for each parameter level, formal inferential statistical analysis (e.g., confidence intervals or hypothesis testing) was not feasible. Instead, the results were treated in a descriptive statistical sense through normalized response indices, percentage changes under controlled comparison groups, and quantitative benchmarking against similar SRC impact tests reported in the literature. This limitation is particularly relevant to the non-monotonic response associated with stirrup spacing, for which the available evidence supports only a balanced-response interpretation within the investigated range, rather than a definitive optimum.

A second source of uncertainty arises from model scale. All specimens were designed as 1:2 scaled columns. This provides a practical basis for controlled impact testing, but it does not fully eliminate scale effects. Local crushing, crack development, interface interaction, and strain-rate sensitivity may not scale in a strictly proportional manner. The present results should therefore be interpreted primarily as revealing section-dependent behavioral trends and relative parameter effects, rather than as direct predictions for full-scale members. Under ideal geometric similarity, the characteristic scales of several global response quantities may be expressed in approximate form, with force, displacement, and impact energy varying with λ^2^, λ, and λ^3^, respectively, where λ is the geometric scale factor. For a gravity-driven drop-weight problem, strict similitude would in principle also require consideration of a Froude-type velocity scale, while inertia-to-stiffness effects may be viewed in relation to a Cauchy-type parameter. The present experimental program was not intended as a strict model–prototype reproduction satisfying full similitude, but rather as a comparative parametric study within a consistent model series. These basic scaling considerations therefore serve only to clarify the interpretive scope of the results, not to support direct quantitative extrapolation to prototype members.

These limitations do not undermine the significance of the experimental observations, but they do define their present scope. Within that scope, the tests provide a consistent basis for comparing the relative influence of the governing parameters and for identifying the main behavioral characteristics of L-shaped SRC columns under lateral impact. In particular, impact velocity emerged as the dominant driver of response severity, axial compression showed a beneficial but bounded effect, and stirrup spacing governed a confinement-related trade-off among resistance, deformation control, and energy dissipation.

From an engineering perspective, the present results are most useful as a basis for preliminary assessment and detailing considerations for asymmetric SRC members subjected to lateral impact. The findings suggest that transverse reinforcement should not be selected solely on the basis of maximizing confinement, and that residual deformation warrants particular attention in L-shaped SRC columns because of its pronounced sensitivity to both impact severity and confinement level. Further work should include repeated tests, expanded specimen matrices, and full-scale or multi-scale validation, together with direct strain-rate measurements and refined numerical modeling, in order to establish more general design-oriented relationships.

## 5. Conclusions

This study experimentally examined the effects of impact velocity (v), axial compression ratio (n), and stirrup spacing (s) in the non-densified region on the failure mode, dynamic response, and energy dissipation of L-shaped steel-reinforced concrete (SRC) columns under lateral drop-weight impact. A total of seven specimens were tested. The main conclusions are as follows.

(1) Within the investigated range, impact velocity was the dominant parameter governing the impact response. For specimens with n = 0.1 and s = 150 mm, increasing v from 7.67 m/s to 9.90 m/s increased Fmax and Fave by 56.3% and 76.6%, respectively. Over the same range, Δmax, Δres, and T increased by 92.6%, 144.3%, and 110.0%, respectively. The absorbed energy E_ab_ increased from 6.06 kJ to 12.38 kJ, and the energy absorption ratio η increased from 60.7% to 74.6%. Higher impact velocity therefore led to greater force demand, larger deformation, more severe damage, and higher energy dissipation.

(2) Within the investigated range (n ≤ 0.2), axial compression had a beneficial effect on resistance enhancement and deformation control. Under v = 8.85 m/s ands = 150 mm, increasing n from 0 to 0.2 increased Fmax and Fave by 17.5% and 30.4%, respectively, while reducing Δmax and Δres by 11.5% and 32.1%. The impact duration T decreased by 28.6%, and η increased by about 6.2 percentage points. These results indicate that moderate axial compression improved plateau resistance and limited post-impact deformation. Under large lateral displacement, however, second-order (P–Δ) effects may become significant and should be considered in high-energy impact design.

(3) The effect of stirrup spacing was non-monotonic. For specimens with v = 7.67 m/s and n = 0.1, increasing s from 100 mm to 200 mm increased Fmax by 12.0% but reduced Fave by 9.6%. Over the same range, Δmax, Δres, and T increased by 52.7%, 42.9%, and 51.6%, respectively. The highest η (60.7%) was obtained at s = 150 mm, whereas the specimen with s = 100 mm showed the smallest deformation demand but a much lower η (38.6%). These results reflect a trade-off among resistance, deformation control, and energy dissipation. Within the investigated range, s = 150 mm provided the most balanced overall response among the tested cases, rather than representing a definitive optimum.

(4) The embedded L-shaped steel section was essential to post-impact integrity. No specimen exhibited global instability or collapse, and no global buckling of the embedded steel section was observed. Even when severe concrete crushing and spalling occurred, the steel section maintained a continuous internal load path and preserved overall member stability. The internal steel skeleton therefore remained the principal load-resisting component after significant local concrete damage.

(5) Within the investigated parameter range, the combination of moderate axial compression (n ≤ 0.2) and intermediate stirrup spacing (s = 150 mm) was associated with the most balanced performance in terms of resistance, deformation control, and energy dissipation. This observation may serve as a useful reference for the preliminary assessment and impact-resistant transverse detailing of asymmetric SRC columns under comparable axial-load conditions. Overall, the present results provide experimental support for the performance assessment and detailing optimization of L-shaped SRC members under lateral impact.

The present study was based on a limited specimen matrix. Although the selected parameter design made it possible to identify the effects of impact velocity, axial compression ratio, and stirrup spacing within the investigated range, the statistical robustness and broader applicability of the observed trends remain limited. Future work should include repeated tests, expanded specimen matrices, and validated numerical analyses to improve the reliability of the conclusions. Further studies should also address post-impact residual load-carrying capacity, longer-term durability issues such as corrosion and fatigue, and SRC members incorporating alternative or recycled cement-based materials, so that impact resistance can be assessed together with serviceability, durability, and sustainability.

## Figures and Tables

**Table 1:** Specimen matrix and design parameters.

Specimen ID	Length, L (mm)	Cross-Section (mm × mm)	Drop Height, H (m)	Impact Velocity, v (m/s)	Axial Compression Ratio, n (–)	Stirrup Spacing, s (mm)	Impact Energy, E (kJ)
v8.85–n0–s150	2000	300 × 300	4.0	8.85	0	150	13.29
v8.85–n0.1–s150	2000	300 × 300	4.0	8.85	0.1	150	13.29
v8.85–n0.2–s150	2000	300 × 300	4.0	8.85	0.2	150	13.29
v7.67–n0.1–s100	2000	300 × 300	3.0	7.67	0.1	100	9.97
v7.67–n0.1–s150	2000	300 × 300	3.0	7.67	0.1	150	9.97
v7.67–n0.1–s200	2000	300 × 300	3.0	7.67	0.1	200	9.97
v9.90–n0.1–s150	2000	300 × 300	5.0	9.90	0.1	150	16.61

Note: Impact velocity v was obtained from the drop height H assuming free fall, $v=\sqrt{2gh}$. The nominal impact energy *E* was calculated as $E=\frac{1}{2}mv^{2}$, where g = 9.81 m/s^2^ and m = 339 kg.

**Table 2:** Mix proportion and mechanical properties of C30 concrete.

Parameter	Water (kg/m^3^)	Cement (kg/m^3^)	Sand (kg/m^3^)	Coarse Aggregate (kg/m^3^)	Specimen Name	*f*_*cu*_ (MPa)		*E_c_* (MPa)
					C30-1	32.9		
C30	184.2	392.8	573.4	1251.7	C30-2	33.5	33.7	3.09 × 10^4^
					C30-3	34.7		

**Table 3:** Mechanical properties of steel materials.

Steel Type	Diameter/Thickness/mm	fy/MPa	fu/MPa	Es/MPa	δs/%
Built-in welded L-shaped steel (Q235B)	6	310.4	413.6	2.0 × 10^5^	22.1
HRB400 longitudinal bars	8	453.4	579.1	2.1 × 10^5^	21.8
HPB300 stirrups	6	396.4	531.7	2.1 × 10^5^	21.5
1 × 7 prestressing strand	15.6	1322.4	1892.6	1.95 × 10^5^	23.1

**Table 4:** Summary of specimen parameters and impact response indices.

Specimen ID	N (kN)	E (kJ)	T (ms)	F_max_ (kN)	F_ave_ (kN)	Δ_max_ (mm)	Δ_res_ (mm)	E_ab_ (kJ)	η(%)
v8.85–n0–s150	0	13.29	18.35	713.49	153.54	35.52	30.26	7.25	54.5
v8.85–n0.1–s150	848.8	13.29	15.23	778.34	162.82	31.91	23.22	7.55	56.8
v8.85–n0.2–s150	1697.6	13.29	13.10	838.62	200.19	31.44	20.54	8.07	60.7
v7.67–n0.1–s100	848.8	9.97	9.48	632.86	142.51	18.42	14.62	3.85	38.6
v7.67–n0.1–s150	848.8	9.97	10.04	698.02	133.51	25.20	15.52	6.06	60.7
v7.67–n0.1–s200	848.8	9.97	14.37	708.85	128.83	28.13	20.90	5.17	51.9
v9.90–n0.1–s150	848.8	16.61	21.08	1091.37	235.84	48.54	37.92	12.38	74.6

Note: N is the axial force applied and maintained prior to impact; E is the nominal impact energy; η is the energy absorption ratio.

**Table 5:** Quantitative benchmarking of section-type sensitivity under matched impact conditions.

Condition	Metric	L-Shaped (This Study)	T-Shaped [33]	Cruciform [3]	Key Implication
v = 7.67 → 9.90 m/s n = 0.1, s = 150 mm	Fmax increase	+56.4%	+32.2%	+56.1%	Higher force sensitivity in L-shaped and cruciform sections
	Δmax increase	+92.6%	+80.2%	+88.0%	Largest Δmax growth in the L-shaped section
	Residual deformation increase	+144.3%	+107.3%	+94.7%	Strongest residual-deformation sensitivity in the L-shaped section
n = 0 → 0.2 v = 8.85 m/s, s = 150 mm	Fmax increase	+17.5%	+72.1%	+13.1%	Strongest axial-load effect on Fmax in the T-shaped section
	Residual deformation reduction	−32.1%	−17.5%	−9.9%	Strongest post-impact deformation reduction in the L-shaped section
s = 100 → 200 mm v = 7.67 m/s, n = 0.1	Δmax increase	+52.7%	+38.5%	+47.9%	Higher confinement sensitivity in the L-shaped section
	η at s = 150 mm	60.7%	NR	49.4%	Higher η at medium spacing in the L-shaped section
Whole test range	η range	38.6–74.6%	NR	33.2–73.9%	η of the L-shaped section was at least comparable to that of the cruciform section

Note: T-shaped data were taken from Xiang et al. [33] (Table 1), and cruciform data were taken from Liu et al. [3] (Table 3). For the cruciform specimens, η was recalculated from Ea/E. T-shaped specimens did not report absorbed energy directly, so η is marked as NR. In the present study, the residual displacement is denoted by Δres, whereas in the T-shaped and cruciform studies the reported value is Δstab. These quantities are treated here as comparable residual-like deformation indices.

## Data Availability

The original contributions presented in this study are included in the article. Further inquiries can be directed to the corresponding authors.

## Appendix A. Fitted Expressions for Normalized Parametric Trends

To support the normalized comparisons presented in Section 4.4, the fitted expressions of the correction functions $\phi_{x}\left(x\right)$ are summarized in this appendix. These expressions were obtained by low-order fitting within each controlled comparison group and are intended only for within-range interpretation of the present test results. They should not be regarded as general predictive equations for design.

For the impact-velocity and axial-compression-ratio groups, specimen v8.85–n0.1–s150 was taken as the reference specimen. For the stirrup-spacing group, specimen v7.67–n0.1–s150 was used as the reference specimen. The normalized response was defined according to Equation (7) in the main text.

Unless otherwise stated, the fitted expressions below describe the trends of normalized $F_{max}$, normalized $\Delta_{max}$, and normalized η with the varying parameter under the corresponding controlled conditions. Owing to the limited specimen matrix and the absence of repeated tests, these expressions should be interpreted as compact empirical representations of the observed trends rather than as standalone constitutive or design models.

For convenience, the varying parameters were recentered as follows:

$$\begin{array}{l} X_{v}=v-8.85\\ X_{n}=\frac{n-0.1}{0.1}\\ X_{s}=\frac{s-150}{50} \end{array},$$

The normalized response is expressed in the general form

(A1) $$\frac{R}{R_{ref}}=1+aX+bX^{2},$$

where *R* denotes the selected response quantity and *R_ref_* is the corresponding value of the reference specimen.

### Appendix A.1. Impact-Velocity Group

For specimens with n = 0.1 and s = 150 mm, the normalized correction functions associated with impact velocity v were fitted as follows:

(A2) $$\phi_{\nu }^{F_{\max}}\left(\nu \right)=\frac{F_{\max}}{F_{\max,ref}}=1+0.2439X_{v}+0.1325X_{v}^{2},$$

(A3) $$\phi_{\nu }^{\Delta_{\max}}\left(\nu \right)=\frac{\Delta_{\max}}{\Delta_{\max,ref}}=1+0.3465X_{v}+0.1427X_{v}^{2},$$

(A4) $$\phi_{\nu }^{\eta }\left(\nu \right)=\frac{\eta_{\max}}{\eta_{ref}}=1+0.1305X_{v}+0.1599X_{v}^{2},$$

These expressions describe the fitted trends of normalized Fmax, Δmax, and η over the tested velocity range. Because specimen v8.85–n0.1–s150 was used as the reference, the normalized η trend should be interpreted with reference to the chosen baseline rather than as evidence of a strictly monotonic absolute increase.

### Appendix A.2. Axial-Compression-Ratio Group

For specimens with v = 8.85v = 8.85v = 8.85 m/s and s = 150 mm, the normalized correction functions associated with axial compression ratio n were fitted as follows:

(A5) $$\phi_{n}^{F_{\max}}\left(n\right)=\frac{F_{\max}}{F_{\max,ref}}=1+0.0804X_{n}-0.0029X_{n}^{2},$$

(A6) $$\phi_{n}^{\Delta_{\max}}\left(n\right)=\frac{\Delta_{\max}}{\Delta_{\max,ref}}=1-0.0639X_{n}+0.0492X_{n}^{2},$$

(A7) $$\phi_{n}^{\eta }\left(\nu \right)=\frac{\eta_{\max}}{\eta_{ref}}=1+0.0546X_{v}+0.0141X_{n}^{2},$$

These expressions indicate that increasing axial compression ratio was associated with a moderate increase in normalized Fmax and normalized η, together with a slight decrease in normalized Δmax, within the investigated range.

### Appendix A.3. Stirrup-Spacing Group

For specimens with v = 7.67 m/s and n = 0.1, the normalized correction functions associated with stirrup spacing s were fitted as follows:

(A8) $$\phi_{s}^{F_{\max}}\left(s\right)=\frac{F_{\max}}{F_{\max,ref}}=1+0.0544X_{n}-0.0389X_{s}^{2},$$

(A9) $$\phi_{s}^{\Delta_{\max}}\left(s\right)=\frac{\Delta_{\max}}{\Delta_{\max,ref}}=1+0.1927X_{s}-0.0764X_{s}^{2},$$

For the normalized energy absorption ratio, the non-monotonic trend was approximated by a quadratic relation in terms of the transformed spacing variable Xs, with Xs = 0 corresponding to s = 150 mm:

(A10) $$\phi_{n}^{\eta }\left(X_{s}\right)=\frac{\eta }{\eta_{ref}}=1+0.1096X_{s}-0.2545X_{s}^{2},$$

This quadratic form was introduced to capture the non-monotonic variation observed at the three tested spacing levels only. Therefore, it should be interpreted as a within-range empirical fit rather than as evidence of a universal optimum stirrup spacing.

### Appendix A.4. Scope and Limitation of the Fitted Expressions

The above expressions were fitted from a limited experimental matrix consisting of only three data points within each controlled comparison group and without repeated tests. They are therefore intended only to provide a compact quantitative description of the observed first-order trends within the investigated range. They should not be interpreted as generalized predictive equations for design, nor should they be extrapolated beyond the tested parameter combinations. This limitation is consistent with the scope stated in Section 4.4.
